# Symbolic Analysis of the Quality of Texts Translated into a Language Preserving Vowel Harmony

**DOI:** 10.3390/e27090984

**Published:** 2025-09-20

**Authors:** Kazuya Hayata

**Affiliations:** Sapporo Gakuin University, Ebetsu 069-8555, Japan; hayata@sgu.ac.jp

**Keywords:** ordinal pattern, binary sequence, Ural-Altaic languages, backtranslation, machine translation, artificial intelligence

## Abstract

To date, the ordinal pattern-based method has been applied to problems in natural and social sciences. We report, for the first time to our knowledge, an attempt to apply this methodology to a topic in the humanities. Specifically, in an effort to investigate the applicability of the methodology in analyzing the quality of texts that are translated into a language preserving the so-called vowel harmony, computed results are presented for the metrics of divergence between the back-translated and the original texts. As a specific language we focus on Japanese, and as metrics the Hellinger distance as well as the chi-square statistic are employed. Here, the former is a typical information-theoretical measure that can be quantified in natural unit, nat for short, while the latter is useful for performing a non-parametric testing of a null hypothesis with a significance level. The methods are applied to three cases: a Japanese novel along with a translated version available, the Preamble to the Constitution of Japan, and seventeen translations of an opening paragraph of a famous American detective story, which include thirteen human and four machine translations using DeepL and Google Translate. Numerical results aptly show unexpectedly high scores of the machine translations, but it still might be too soon to speculate on their unconditional potentialities. Both our attempt and results are not only novel but are also expected to make a contribution toward an interdisciplinary study between physics and linguistics.

## 1. Introduction

In addition to several explicit rules, speakers all over the world are implicitly bound by a sound pattern of their own native languages. For instance, we consider the sound arrangement of a polysyllabic word. Extracting vowels from, for instance, a trisyllabic word makes possible five arrangements: ABC, AAB, ABA, ABB, and AAA, where symbols A, B, and C represent a vowel in a sound system that is inherent in each individual language. For typical European languages, irrespective of the parts of speech, the pattern of ABC is dominant [1], in contrast to the dominance of AAA in, e.g., Mongolian [2]. In linguistics, it is well known that languages belonging to the Ural–Altaic languages and some languages belonging to the Austronesian as well as the Bantu family, in general, preserve the ‘vowel harmony’ [3]. This phenomenon describes a phonetic rule that prescribes the coexistence of similar vowels, such as /u, o/ and /i, e/, in a polysyllabic word. Owing to the rule, in addition to the abovementioned Mongolian, Japanese nouns tend to prefer AAB, in contrast to the preference of ABB in such languages as Indonesian, Telugu, and Ainu [2]. Of them, the sound pattern of Japanese texts appears unique in that the lexicon consists of three strata [4]; that is, native words [5], loan words of Chinese origin, and those of European origin [4]. (Incidentally, this model might bear a remote resemblance to a useful model to document the expansion of English today, developed by an Indian–American linguist, Braj B. Kachu, who employs three concentric circles to reflect the different ways in which English continues to gain new speakers [6].) Here, we note that native words prefer the conventional pattern AAB and AAA, whereas both loan words prefer ABC and ABA to the other three [2]; examples of the former (AAB and AAA) are *kusuri* ‘medicine’ and *kokoro* ‘mind,’ while those of the latter (ABC and ABA) are *kazoku* ‘family’ and *karuta* ‘card.’ This historical blend of the five vowel arrangements results in a golden mean between the archaic Japanese and the modern counterpart that has been perturbed by Chinese, Portuguese, Dutch, German, French, and English.

We here attempt to investigate the applicability of the ordinal pattern-based approach [7,8,9,10,11,12,13,14,15,16,17,18,19,20,21,22,23,24,25,26,27,28,29,30,31,32,33,34,35,36] to evaluating the quality of texts that are translated into a language preserving the vowel harmony. To our knowledge, this powerful methodology has been applied to topics solely in the natural as well as social science. Historically the mainstream attempts were seen in the chaotic symbolic mapping [9,17,25,31] and in the physiological signal processing [13,19,20,22,24,26,27,32,34]. Subsequently, the method has been successfully applied to diverse areas of science that include laser physics [18,30,33] and economics [16,20,23]. More recently it has found novel applications to climatology [15,28], space physics [29], flight delay analysis [35], and hydrology [36]. Despite the sustained efforts to investigate the applicability of the ordinal pattern-based approach, however, it seems that no attempt has been made to explore problems in the humanities. Here, we report, for the first time to our knowledge, an attempt to apply the ordinal pattern-based statistics to an interesting topic in linguistics that belongs to the humanities and, at the same time, bears a connection with the other branches of science. Computed results are presented for the metrics of divergence between the back-translated and the original texts. As a specific language, we focus on Japanese, and as metrics, the Hellinger distance as well as the chi-square statistic are employed. The former is a typical information-theoretical quantity that is measured in natural unit, nat for short [28], while the latter is useful for performing non-parametric testing with a significance level [1,2,5]. The methods are applied to three cases: a Japanese novel written by Kenzaburo Oe (1935–2023) [37,38], a Japanese Nobel laureate for literature in 1994, the Preamble to the Constitution of Japan [39], and seventeen translations of the opening paragraph of a detective story written by Edgar Allan Poe (1809–1849) [40], which include thirteen human [41,42,43,44,45,46,47,48,49,50,51,52,53] and four machine translations using DeepL and Google Translate. A detailed analysis of the last case demonstrates that of the seventeen translations, in only two human translations as well as in one machine translation the style is far from stochastic with the significance level 0.1% and 5%, respectively. To our knowledge, this observation on the broken symmetry is novel and, at the same time, can be expected to have a nontrivial impact on the interdisciplinary field between physics and linguistics. A diachronic analysis of results from human and machine translations is expected to reveal a potential of an updated machine translation by a specific artificial intelligence utilizing a statistically based program [54].

## 2. Procedure of Binary Coding

**Figure 1:**
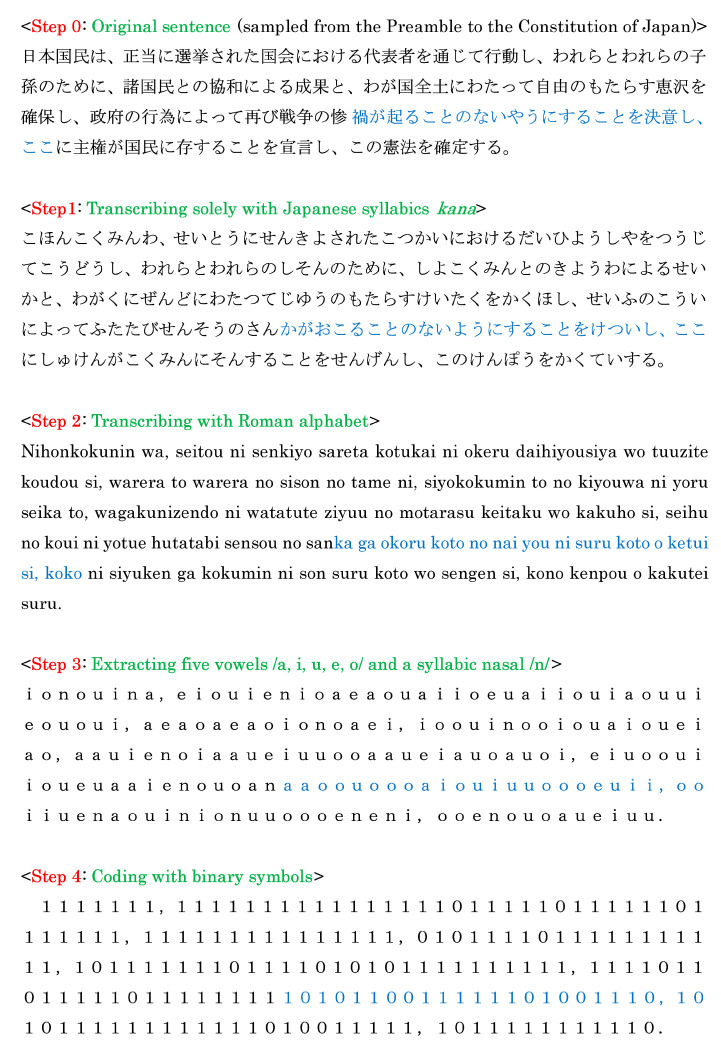
Process of generating binary sequence. The binary sequence in the final step is generated according to the rule of Equation (1). The sequence highlighted in blue will be mentioned in Figure 2.

The procedure necessary for generating a binary sequence from a Japanese text is shown in Figure 1. First, the original text written with the amalgam of Chinese characters and Japanese syllabics, *kana*, is transcribed only with syllabics (Step 1), followed by transcribing the syllabic sequence with the Roman alphabet (Step 2). Subsequently, the six sounds consisting of the five vowels /a, i, u, e, o/ as well as a syllabic nasal /n/ are extracted from the latter sequence (Step 3). Finally, the binary sequence *s*_1_*s*_2_ … *s_n_* can be obtained according to the rule (Step 4)

**Figure 2:**
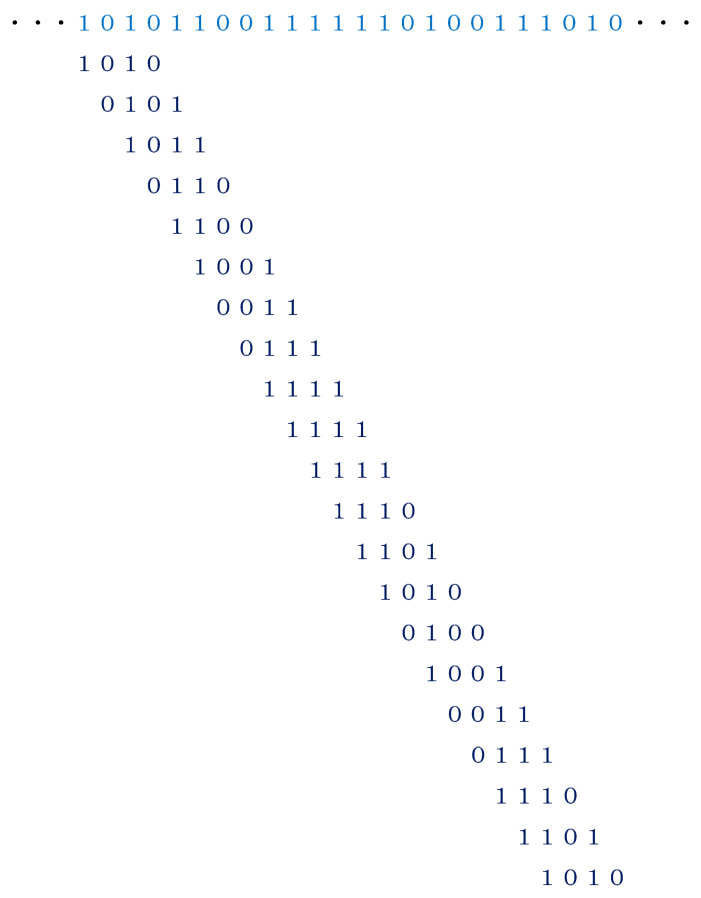
An example for explaining how to generate 4-bit binary codes from data highlighted in blue for Step 4 of Figure 1. Punctuation marks in Figure 1 are dropped.

*s_i_* = 0, if *x_i_*_+1_ coincides with *x_i_*; *s_i_* = 1, otherwise, (1)

for *i* = 1, 2, …, *n*, where *x_i_* (*i* = 1, 2, …, *n*) represents one of the six sounds in Step 3, and *n* indicates the length of the binary sequence. An example for explaining how to generate 4-bit binary codes from data that are highlighted in blue for Step 4 is given in Figure 2. Eventually, one can produce *n* − 3 binary codes from the sound sequence of Step 3. With the definition of the 16 binary codes,

*C*_1_ = 0000, *C*_2_ = 0001, *C*_3_ = 0010, *C*_4_ = 0011,      *C*_5_ = 0100, *C*_6_ = 0101, *C*_7_ = 0110, *C*_8_ = 0111,         *C*_9_ = 1000, *C*_10_ = 1001, *C*_11_ = 1010, *C*_12_ = 1011,            *C*_13_ = 1100, *C*_14_ = 1101, *C*_15_ = 1110, *C*_16_ = 1111, (2)

the 21 codes in Figure 2 can be written as

C11C6C12C7C13C10C4C8C16C16C16C15C14C11C5C10C4C8C15C14C11.

## 3. Metrics of Divergence

To evaluate the quality of translated texts, the Hellinger distance is useful:

(3) $$D_{H\;}(nat)\;=\;\sum_{i\;=1}^{m}(\sqrt{p_{i}}-\;\sqrt{q_{i}\;}{)}^{2},\;\;$$

where *p_i_* and *q_i_* (*i* = 1 to *m*) represent the relative frequencies of *C_i_*, (see Equation (2) for *m* = 16) in the two sequences to be compared; *m* denotes the number of categories of the code (for the 4-bit coding, *m* = 16). In order to conduct statistical testing, the chi-square value is used:

(4) $$\chi^{2}\;=\;\sum_{i\;=1}^{m}\frac{(f_{i}-F_{i}{)}^{2}}{F_{i}}.\;\;\;\;\;$$

Here, *f*_i_ and *F*_i_ (*i* = 1 to *m*) represent the surveyed and expected frequencies, respectively. Note that *F_i_* ≠ 0 unless *f_i_* vanishes. The relations between the relative frequencies in Equation (3) and the frequencies in Equation (4) are *p_i_* = *f_i_*/ (*n* – 3) and *q_i_* = *F_i_*/ (*n* – 3). The specific choices of the surveyed and expected frequencies will be given in the following section.

## 4. Results

### 4.1. Passages from a Japanese Novel

First, we consider the backtranslation of passages from *Shiiku* [37] “The Catch” [38], a short novel written by Kenzaburo Oe (1935–2023), the Nobel laureate for literature in 1994. Note that for this work, he was awarded the Akutagawa Prize in 1958, as an eminent young writer. The published translation into English [38] is given in Appendix A.

**Figure 3:**
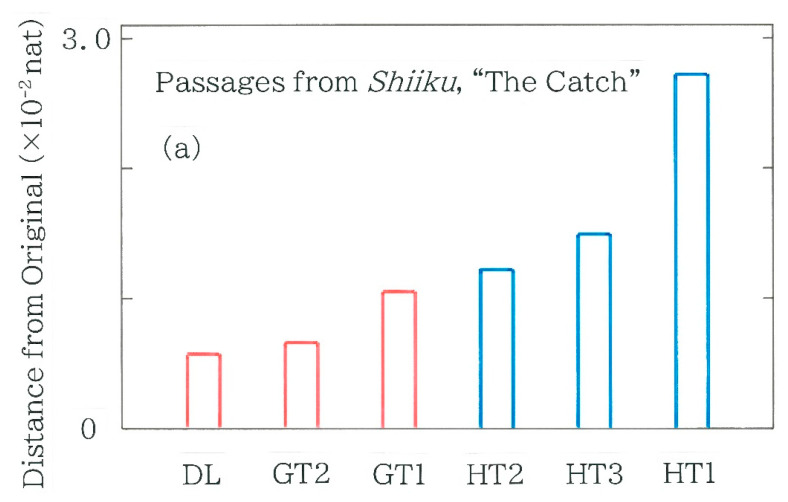

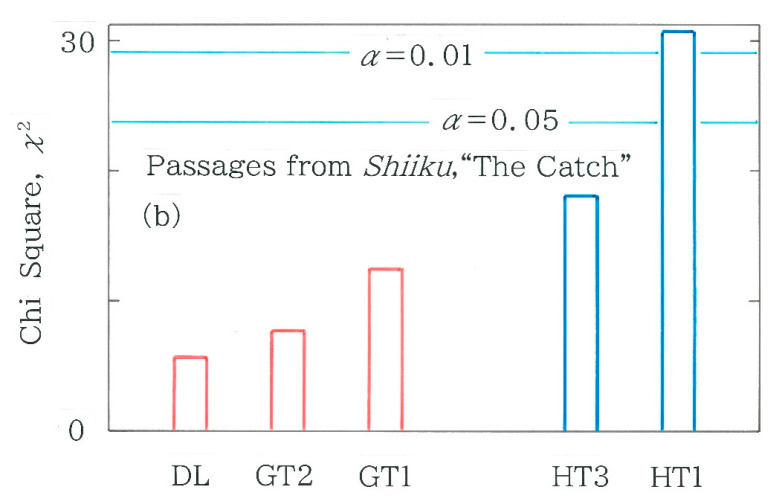
Metrics of divergence between the backtranslation and the Japanese original of passages from *Shiiku*, “The Catch,” by Kenzaburo Oe. The bars enclosed in red and those in blue indicate the machine backtranslations using DeepL (DL) and Google Translate (GT1 and GT2) and the human backtranslations (HT1, HT2, and HT3), respectively. The length of the binary sequence, *n*, is 324, 320, 345, 357, 356, and 394, respectively, while that of the Japanese original is 330. (**a**) Hellinger distance (Equation (3)). (**b**) Chi-square (Equation (4)). The Greek letter *α* denotes the significance level.

Computed results of the Hellinger distance, Equation (3), from the Japanese original [37] are shown in Figure 3a, where the bars enclosed in the red lines (henceforth, for short, ‘red bars’) indicate the machine backtranslations (i.e., from English [38] into Japanese) using DeepL, DL for short (as of July 2025), and Google Translate [54], GT1 (as of April 2020) and GT2 (as of October 2024), while the bars enclosed in the blue lines (henceforth, for short, ‘blue bars’) indicate the backtranslations [55] by HT1 (a male translator in his thirties), HT2 (a female translator in her early forties), and HT3 (a female translator in her forties). It is interesting to note in Figure 3a that independent of the device the distances of the three machine translations are smaller than those of the three human translations. Furthermore, one can see a negative correlation of the distance with the date of machine translation. It can be seen that except for HT1 the distance grows gradually in order from left to right; at HT1 it increases substantially. To inspect the discontinuity in more detail, calculations have been made for the chi-square values being defined in Equation (4), the results of which are shown in Figure 3b. Here, the surveyed and the expected frequencies in Equation (4) are obtained from the frequency distribution of the back-translated and the original text [37], respectively. Note that the bar of HT2 becomes blank because for HT2 a singularity that arose on *C*_1_: 0000 makes impossible the calculation of the chi-square, where the divergent term (3 − 0)^2^/0 was included in the calculation of Equation (4). A positive correlation is observed with the results in Figure 3a. It is found from the plots of Figure 3b that except for HT1 the null hypothesis cannot be rejected (*α =* 0.05), whereas for HT1 the hypothesis is rejected (*α =* 0.01).

Although it could not be assumed that our quantitative measure alone can completely capture the quality of translations, our method based on the ordinal pattern can reveal how faithfully each individual backtranslation reproduces the binary pattern of the original. Finally, it should be noted that the reduced chi-square for GT2 (as of October 2024) in comparison with GT1 (as of April 2020) shows coherence with the improvement in the stylistic quality of the translated text. That is, careful inspection in the backtranslation by GT1 has revealed several flaws, such as a few rudimental errors in Japanese grammar on the use of an active and a passive voice, an excessive repeat of a specific particle, and a rash transliteration instead of translation. In contrast to the backtranslation by GT1, we can find considerable improvement in the style of GT2.

### 4.2. The Preamble to the Constitution of Japan

Next, we consider the Japanese version of the Preamble to the Constitution of Japan. The preamble declares the reason, aim, idea, and principle of the constitution that was promulgated on 3 November 1946 and came into effect on 3 May 1947. Owing to a complicated historical reason in the chaos directly after the end of the Second World War, there are two versions of the constitution available: Japanese and English. The entire preamble of the latter is given in Appendix B. The aim of this section is to investigate the ability of currently available machine translations through comparison between the original Japanese text and those translated from the English version into Japanese. Machine translation devices, DeepL and Google Translate, are employed, along with a human translation by Kayoko Ikeda [39].

**Figure 4:**
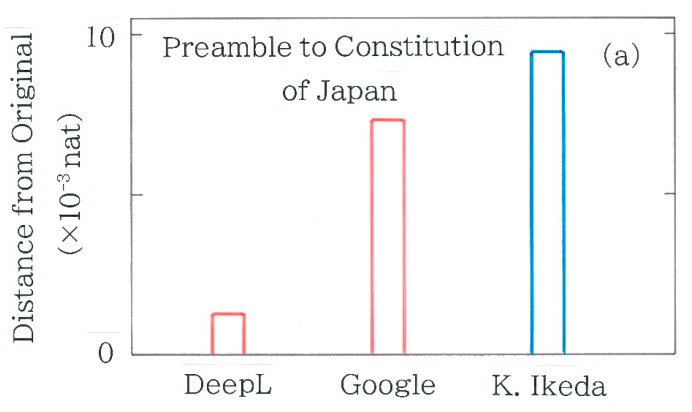

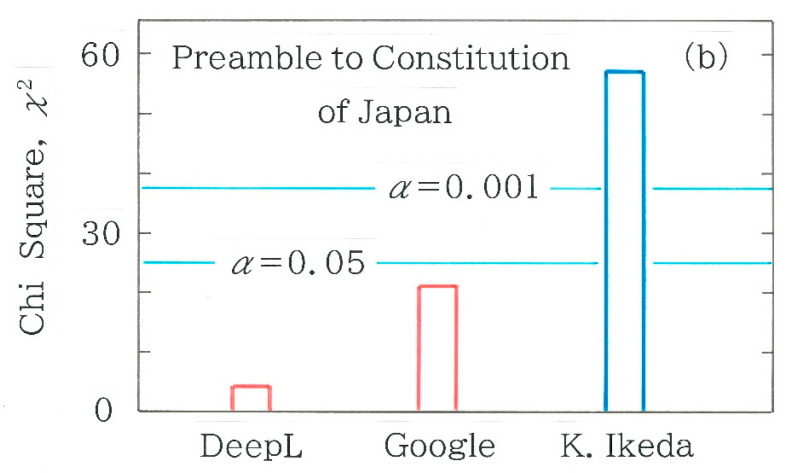
Metrics of divergence between the backtranslation and the Japanese original of the Preamble to the Constitution of Japan. Red and blue bars indicate the machine backtranslations using DeepL and Google Translate and the human backtranslation by Kayoko Ikeda, respectively. The length of the binary sequence, *n*, is 809, 840, and 922, respectively, while that of the Japanese original is 818. (**a**) Hellinger distance. (**b**) Chi-square. The Greek letter *α* denotes the significance level.

In Figure 4a, computed results are shown of the Hellinger distances between the backtranslation into Japanese and the Japanese original of the Preamble to the Constitution of Japan. Here, the red bars indicate machine backtranslations (as of July 2025) using both DeepL and Google Translate, while the blue bars indicate the human backtranslation by Ikeda [39]. Again, it is found that the distances of the machine translations are smaller than that of the human. Indeed, prior to the computation the largest distance of Ikeda’s translation was anticipated because, being inspired by the translated lyrics of *Imagine*, a popular song by John Winston Ono Lennon (1940–1980), she had decided to publish her own translation of the constitution, in an effort to rewrite the original text as concisely as possible. Subsequently, the results of the chi-square values are shown in Figure 4b. Here, the surveyed and the expected frequencies in Equation (4) are obtained from the frequency distribution of the back-translated and the original Japanese text, respectively. For both machine translations the null hypothesis is not rejectable (*α =* 0.05), whereas for the human translation it is rejected (*α =* 0.001).

### 4.3. Passages from an English Story

While the above two cases have dealt with the backtranslations into Japanese, in what follows we consider the translation of an English text into Japanese. Specifically, we focus on the opening paragraph in *The Fall of the House of Usher* [40], a short story written by Edgar Allan Poe (1809–1849), an American mystery writer and poet regarded as a pioneer of detective stories. To our knowledge, this short story has been translated 13 times into Japanese over the past 95 years [41,42,43,44,45,46,47,48,49,50,51,52,53]. The original English text [40] is given in Appendix C.

**Figure 5:**
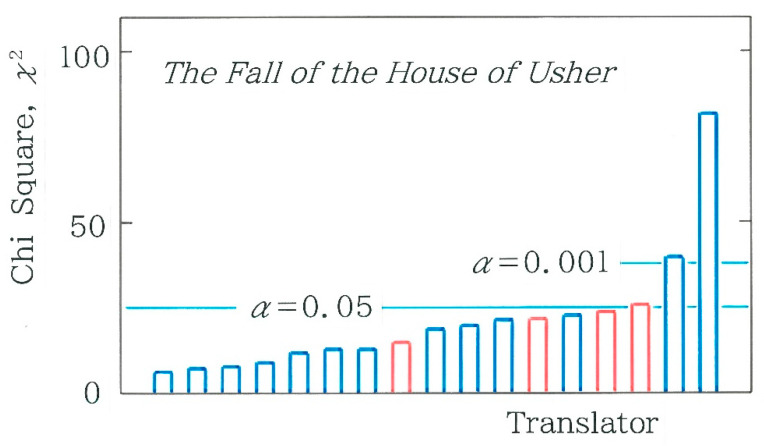
Comparison among the chi-square values for 17 Japanese translations of the opening paragraph in *The Fall of the House of Usher* written by Edgar Allan Poe. The blue and red bars indicate the human and machine translations, respectively. The length of the sequence, *n*, is, in order from the left to right, 1216, 1084, 1160, 1328, 1131, 1037, 1098, 1133, 1194, 1280, 1317, 1074, 1315, 960, 1017, 1426, and 1216, respectively. The expected frequencies are calculated using Equation (4) along with Equations (5a)–(5f).

In Figure 5, comparison is made among chi-square values for 17 Japanese translations of the opening paragraph. The blue and red bars indicate the human and machine translations, respectively. In applying Equation (4) the expected frequencies *F_i_* (*i* = 1 to 16) can be obtained with the blend of ‘0’ and ‘1’ in the entire binary sequence:

(5a) F1 =1DM4, 

(5b) Fi=14DM3N−M1      for i=2, 3, 5, 9, 

(5c) Fi=16DM2N−M2     for i=4, 6, 7, 10, 11, 13, 

(5d) Fi=14DM1N−M3        for i=8, 12, 14, 15, 

(5e) F16=1DN−M4, 

(5f) D=N4.

Here, *M* and *N*, respectively, are the total of ‘0’ and the grand total of ‘0’ and ‘1’. Note that for the 4-bit coding *N* = 4 (*n* – 3). As for the machine translations (red bars) in Figure 5, in order from the left to the right, one can see Google Translate 1 (GT1 in short; as of December 2023; *χ*^2^ = 14.957), GT2 (as of May 2025; *χ*^2^ = 21.735), DeepL (as of July 2025; *χ*^2^ = 23.936), and GT3 (as of July 2025; *χ*^2^ = 25.517). It is interesting to note that the three bars of GT2, DeepL, and GT3 exhibit a height comparable to each other, but solely for GT3 the null hypothesis is rejected (*α* = 0.05). As for the human translations (blue bars), of the 13 translations, only 2 on the right extreme, which correspond to Matsumura [42] (*χ*^2^ = 40.308) and Maruya [50] (*χ*^2^ = 81.610), are found to be statistically significant (*α* = 0.001). It should be stressed here that Saiichi Maruya (1925–2012), who received a Cultural Medal in 2011, is well known not only as a translator of *Ulysses* by James Joyce (1882–1941) but also as a writer of his unique rules for *kana* usage.

**Figure 6:**
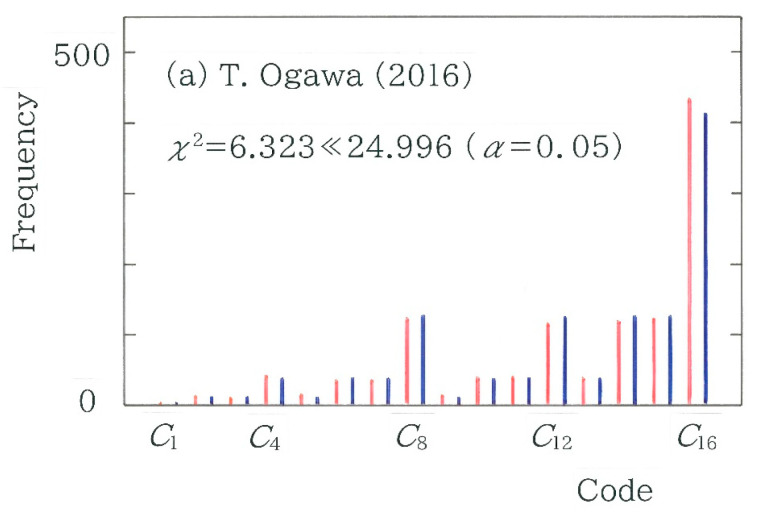

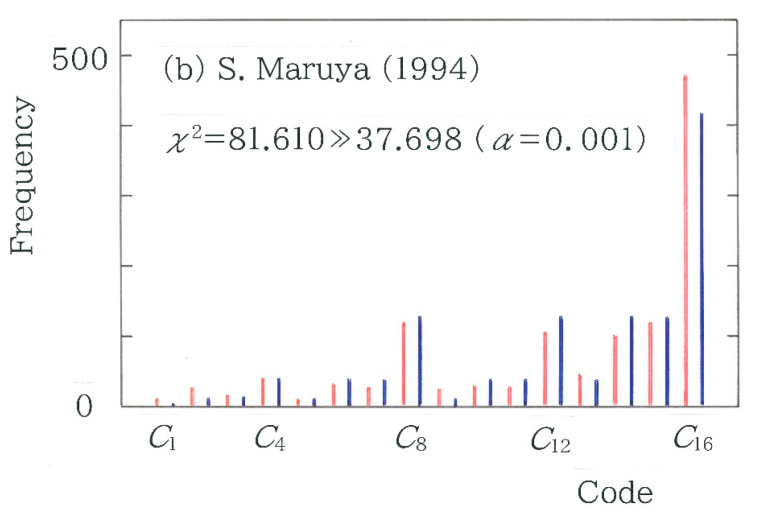
Frequency distributions of the 4-bit binary codes. The red and navy bars indicate the surveyed and expected frequencies, respectively. (**a**) Translation by Takayoshi Ogawa [53]. (**b**) Translation by Saiichi Maruya [50].

To explicitly visualize the difference between the surveyed and expected frequency, in Figure 6, frequency distributions of the 4-bit binary codes *C_i_* (*i* = 1 to 16) are compared with red (surveyed) and navy (expected). Here, from Figure 5 the two bars on the left and right extremes are chosen: (a) Takayoshi Ogawa [53] (*χ*^2^ = 6.323; *n* = 1216) and (b) Saiichi Maruya [50] (*χ*^2^ = 81.610; *n* = 1216). Evidently, it is found that the difference in the topography is consistent with that of the chi-square value. That is, in Figure 6b the sequence of the twin bars consisting of red and navy exhibits a rough topography in particular on the right, which shows a sharp contrast to the relatively smooth topographic configuration being observed in Figure 6a.

## 5. Discussion

### 5.1. Backtranslation Experiment of Ikeda’s Original

To inspect the potential ability of the machine translation, a backtranslation experiment [55] for the Japanese text that was translated by Ikeda [39] from the English version of the Preamble of the Constitution of Japan has been carried out using both DeepL and Google Translate. The experimental procedure is given in the following steps: (1) initially, we start from the Japanese text (assuming J_I_) translated by Ikeda [39]; (2) subsequently, using machine translation devices we translate Text J_I_ into English (assuming E_DL_ for DeepL and E_GT_ for Google Translate); (3) finally, we back-translate Text E_DL_ and E_GT_ into Japanese (assuming J_DL_ and J_GT_, respectively); (4) eventually, we can obtain a chain of the serial translations: Text J_I_→E_DL_→J_DL_ for using DeepL and Text J_I_→E_GT_→J_GT_ for using Google Translate.

**Figure 7:**
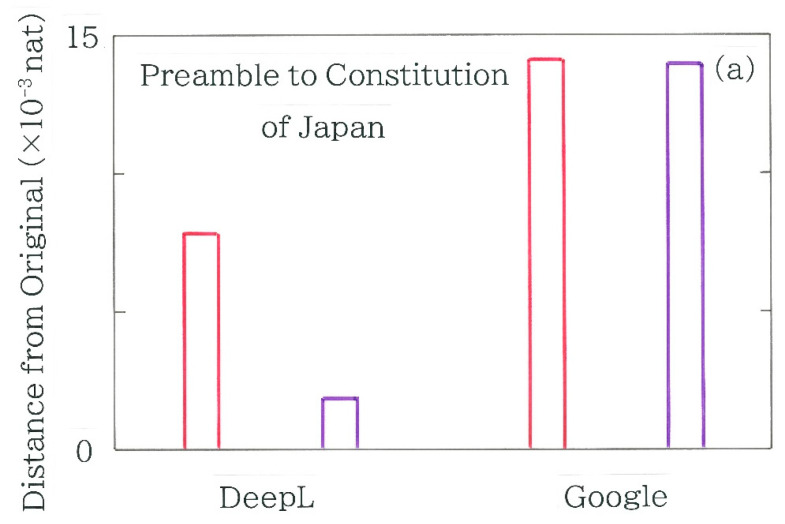

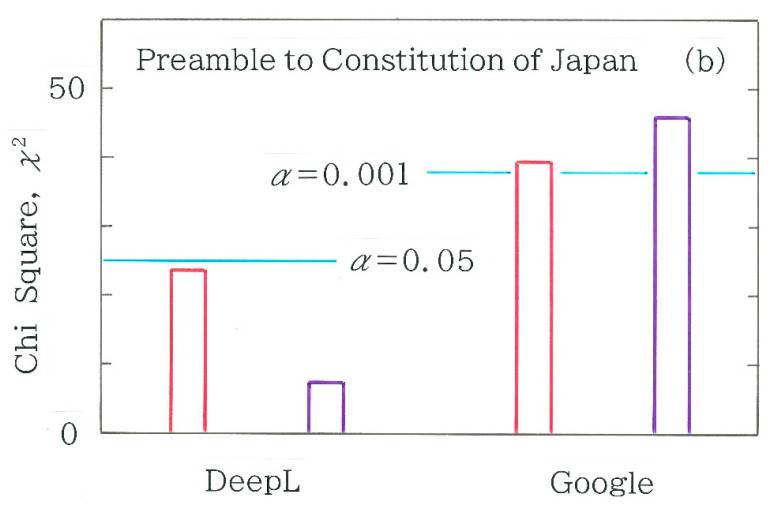
Experimental results of the Hellinger distance and the chi-square value, where the bars enclosed in crimson indicate the divergence from Ikeda’s original [39], while those enclosed in purple indicate the one from the authentic original, i.e., the Japanese original version of the Preamble to the Constitution of Japan. The length of the binary sequence, *n*, is 902 and 940 for DeepL and Google Translate, respectively, while that of the original is 818 and 922 for the authentic and Ikeda’s text, respectively. (**a**) Hellinger distance. (**b**) Chi-square. The Greek letter *α* denotes the significance level.

Experimental results of the Hellinger distance and the chi-square value are shown in Figure 7a,b, respectively, where the bars enclosed in crimson indicate the divergence from Ikeda’s original Text J_I_, while those enclosed in purple indicate the one from the authentic original, i.e., the Japanese version of the Preamble to the Constitution of Japan. It is found that for both Japanese originals the metrics of DeepL become considerably smaller than those of Google Translate. The results of Figure 7b show that the differences between Text J_DL_ and the originals are not statistically significant (*α* = 0.05), whereas the ones between Text J_GT_ and the originals are significant (*α* = 0.001).

### 5.2. Scattergrams for Passages from the Work by Poe

**Figure 8:**
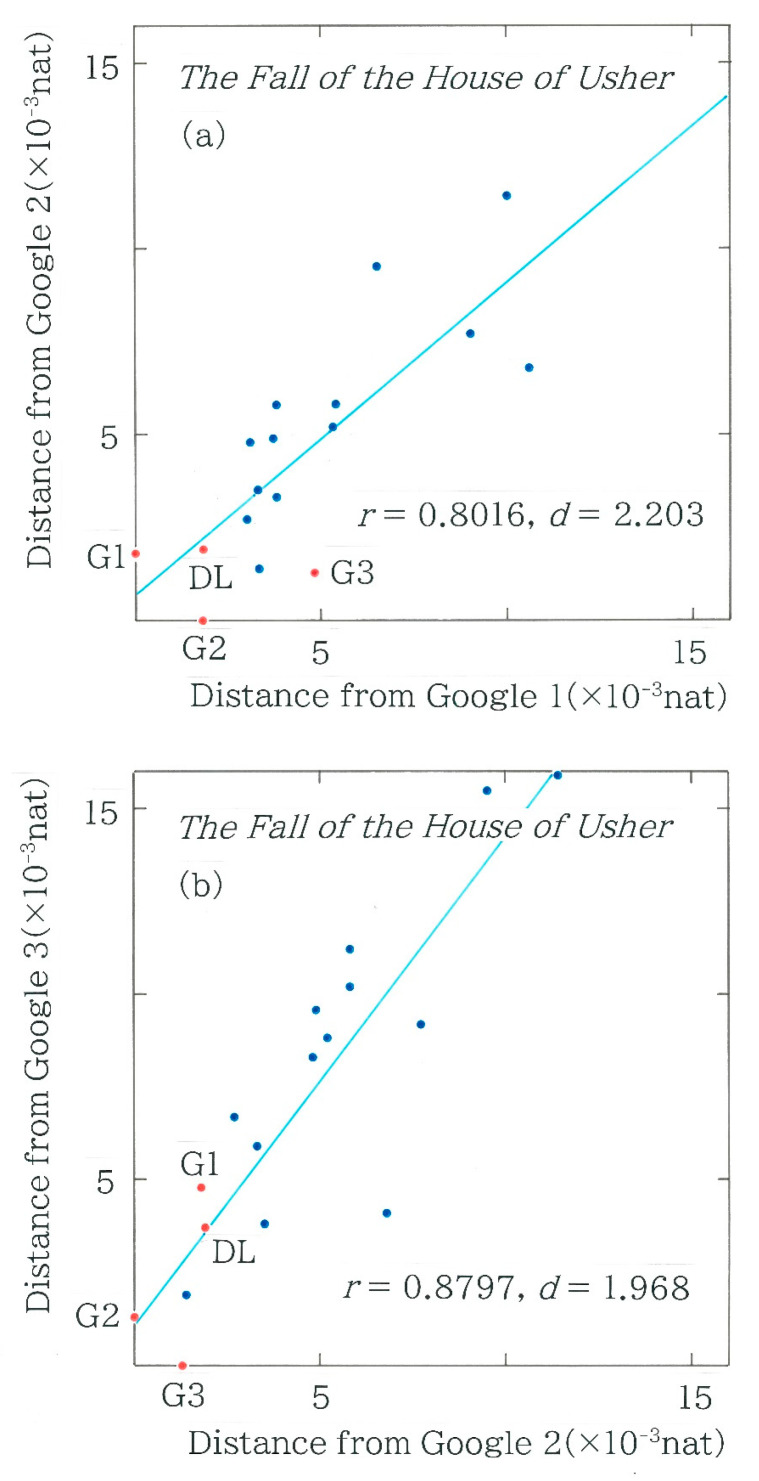

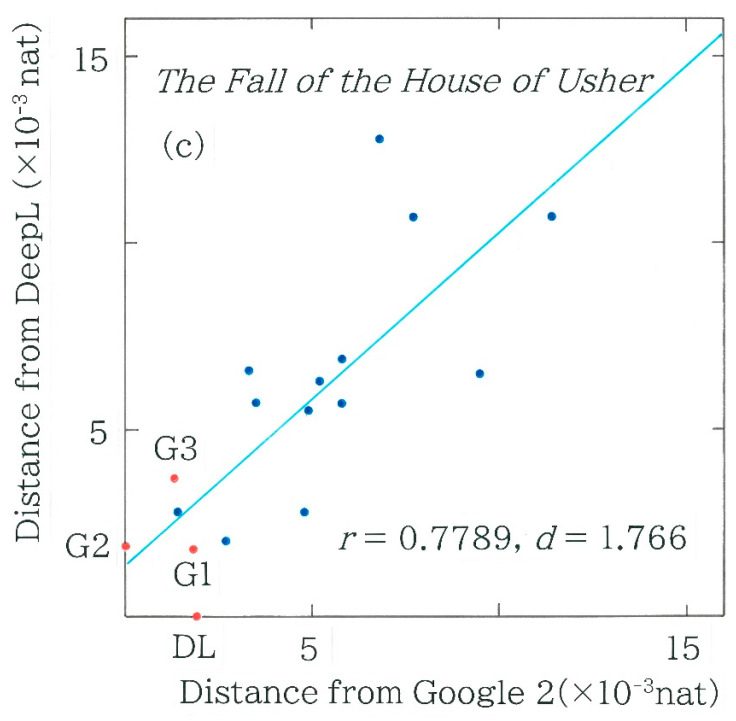
The relation between two Hellinger distances for Japanese translations of the passages from *The Fall of the House of Usher* by Edgar Allan Poe. The blue and red dots indicate the human and machine translations, respectively. The letters *r* (|*r*| ≤ 1) and *d* (0 ≤ *d* ≤ 4) denote Pearson’s correlation coefficient and the Durbin–Watson radio, respectively. The acronyms DL and G indicate DeepL (as of May 2025) and Google Translate, respectively; the number attached to G specifies the date of each machine translation: G1 (as of December 2023), G2 (as of January 2025), and G3 (as of July 2025). (**a**) Hellinger distance from G2 versus that from G1. (**b**) Hellinger distance from G3 versus that from G2. (**c**) Hellinger distance from DL versus that from G2.

To conduct a diachronic analysis of the machine translations, in Figure 8 the relation is plotted between the two Hellinger distances for Japanese translations of the passages from *The Fall of the House of Usher* by Edgar Allan Poe [40]. The acronyms DL and G indicate DeepL (as of May 2025) and Google Translate, respectively; the number attached to G specifies the date of each machine translation: G1 (as of December 2023), G2 (as of January 2025), and G3 (as of July 2025). It can be seen that irrespective of the translation date, there is a moderate positive correlation (0.78 < *r* < 0.88) between the two distances, and of the 13 human dots in blue, the results of Tanizaki [41] are sited in the nearest region from the origin (0, 0). In particular, in Figure 8b the radius of this translator from the origin is much smaller than that of one of Google Translate (G1) as well as DeepL (DL).

**Figure 9:**
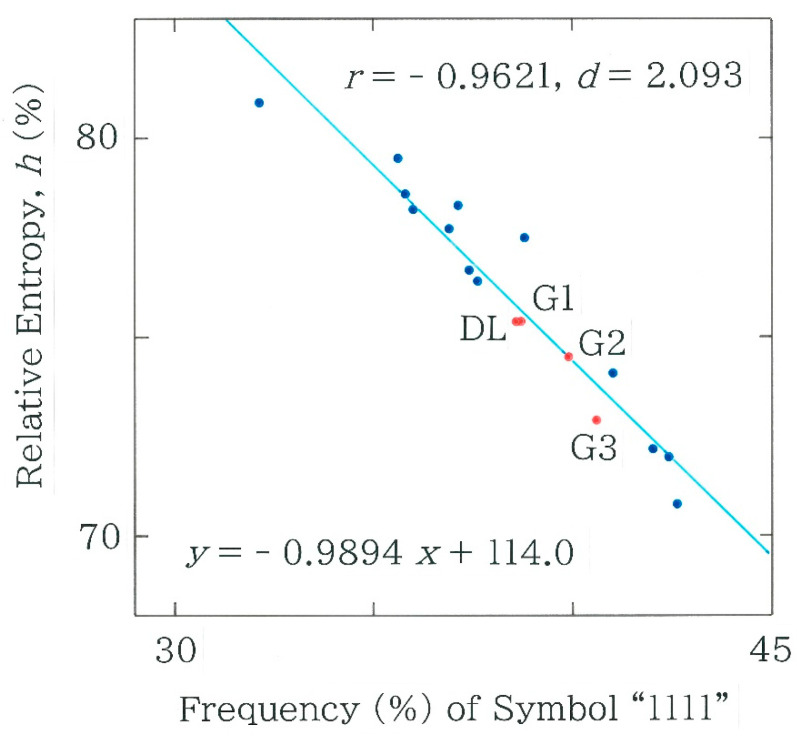
Dependence of the relative entropy (Equation (6)) on the frequency of *C*_16_ “1111.” The results of the human translations [41,42,43,44,45,46,47,48,49,50,51,52,53] are plotted with blue dots, while those of the machine translations with red dots. The acronyms DL and G*i* (*i* = 1, 2, and 3) denote DeepL and Google Translate, respectively.

It should be remembered here that the frequency distributions being plotted in Figure 6 have suggested the dominance of *C*_16_ “1111.” To investigate the results in more detail, in Figure 9 the dependence of the relative entropy *h* (0 ≤ *h* ≤ 1) is shown on the relative frequency of *C*_16_ “1111.” Here, the former is defined by

(6) $$h=-\frac{\sum_{i=1}^{16}p_{i}log{\;p}_{i}}{4\;log2}.$$

In the scattergram of Figure 9 a strong negative correlation (*r* = − 0.9621 with *d* = 2.093) is observed between the entropy and the frequency of *C*_16_ “1111.” The results of the machine translations (DL, G1, G2, and G3) cluster around a centroid on *h* = 0.746 (74.6%). The dot at the upper-left extreme corresponds to the result of Sasaki [45] while the one at the lower-right counterpart to Ooka [51].

### 5.3. Making Investigation into Other Choices of Binary Numbers

Throughout this paper we have concentrated on the 4-bit binary coding because this code length provides the upper limit for the lengths of our binary sequences with 310 < *n* < 1430 (Figure 3, Figure 4, Figure 5, Figure 6 and Figure 7). In other words, for our examples the chi-square testing is not applicable to the coding longer than four. In this section, computed results will be given for the 3-bit coding to investigate the robustness of our results for the 4-bit coding. First, we will revisit the results of Figure 3b for the 4-bit coding. For the 3-bit coding being made we have obtained *χ*^2^ = 2.372 (for HI2), 2.948 (DL), 3.286 (GT2), 6.248 (GT1), 6.734 (HI3), and 11.694 (HI1). Here, the critical chi-square value for *α* = 0.05 is 14.067. Note that the value of HI2, which was blank in Figure 3b, shows the value smaller than that of DL, but for the other five the ranking of the chi-square value remains the same as that seen in Figure 3b. Similar calculations on Figure 4b using the 3-bit coding have shown *χ*^2^ = 1.665 (for DeepL), 11.786 (Ikeda), and 14.245 (Google). Note that although the ranking changes places between Google and Ikeda, the results of the 3-bit coding share, with those of the 4-bit counterpart, the fact that the chi-square value for DeepL becomes substantially smaller than those of the other two.

**Figure 10:**
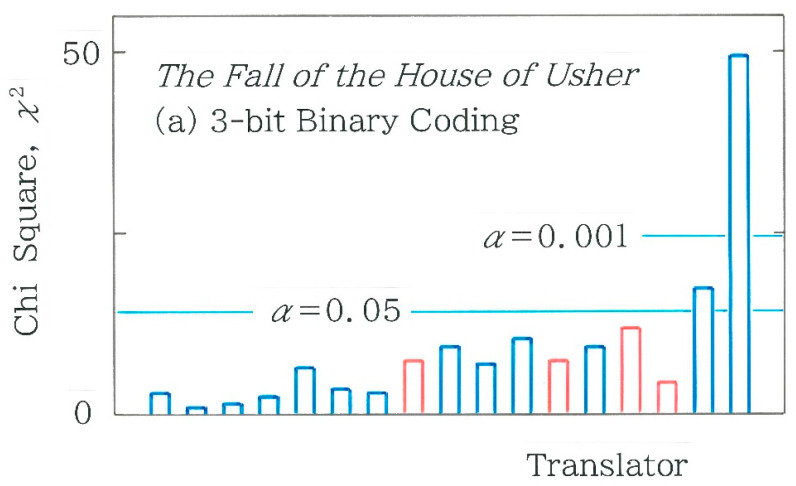

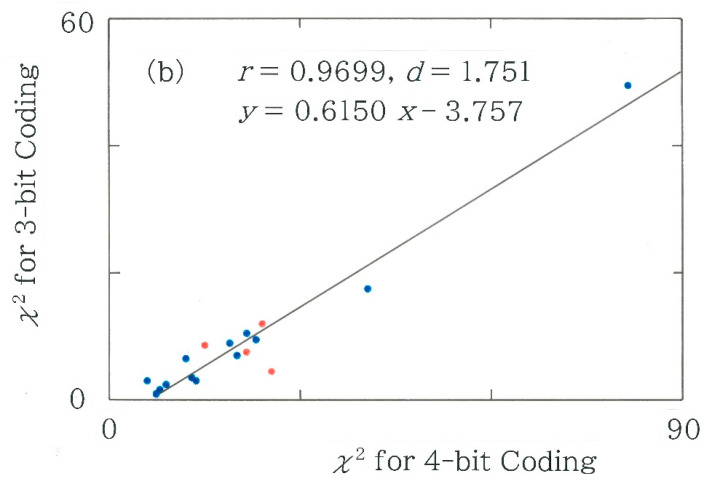
(**a**) Comparison among the chi-square values for 17 Japanese translations of the opening paragraph in *The Fall of the House of Usher* written by Edgar Allan Poe. The blue and red bars indicate the human and machine translations, respectively. The expected frequencies are calculated using Equation (4) along with Equations (7a)–(7e). (**b**) Scattergram of chi-square values for 3-bit coding versus those for 4-bit coding. The blue and red dots indicate the human and machine translations, respectively.

**Figure 11:**
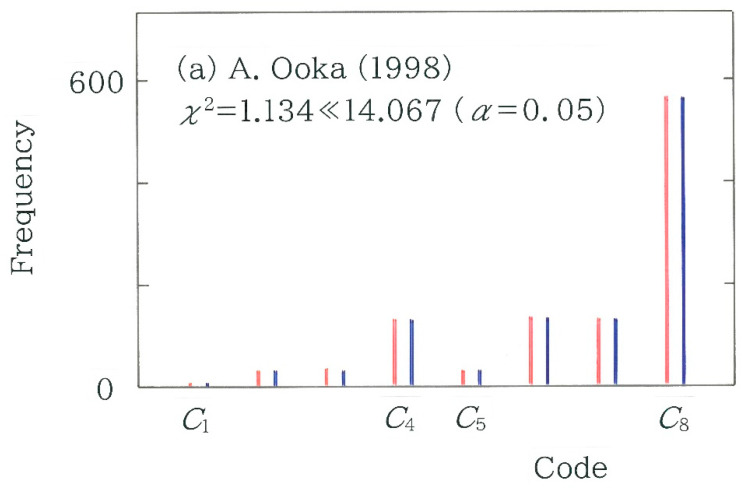

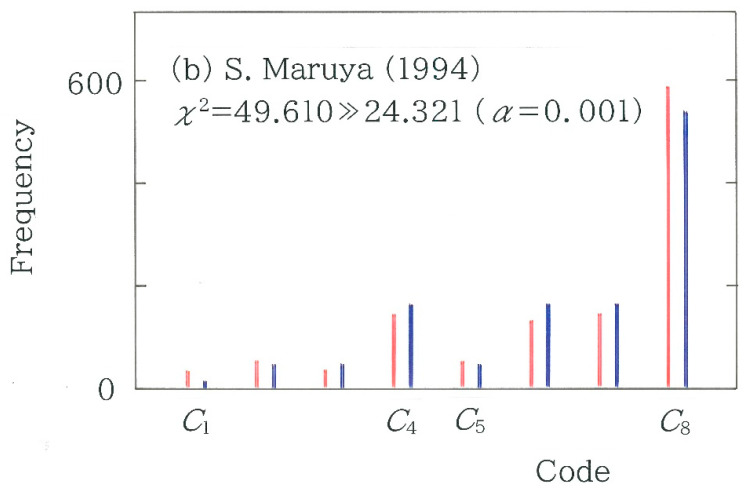
Frequency distributions of the 3-bit binary codes: *C*_1_ = 000, *C*_2_ = 001, *C*_3_ = 010, *C*_4_ = 011, *C*_5_ = 100, *C*_6_ = 101, *C*_7_ = 110, and *C*_8_ = 111. The red and navy bars indicate the surveyed and expected frequencies, respectively. (**a**) Translation by Akira Ooka [51]. (**b**) Translation by Saiichi Maruya [50].

Subsequently, we will revisit the results of Figure 5, where for the 4-bit coding, comparison was made among chi-square values for 17 Japanese translations of the opening paragraph. The results of the 3-bit coding are shown in Figure 10a. Here, the blue and red bars indicate the human and machine translations, respectively. In applying Equation (4) with *m* = 8, instead of Equation (5), the expected frequencies *F_i_* (*i* = 1 to 8) for *C*_1_ = 000, *C*_2_ = 001, *C*_3_ = 010, *C*_4_ = 011, *C*_5_ = 100, *C*_6_ = 101, *C*_7_ = 110, and *C*_8_ = 111 are obtained with the mixture of ‘0’ and ‘1’ in the entire binary sequence:

(7a) F1 =1DM3,        

(7b) Fi=13DM2N−M1      for i=2, 3, 5, 

(7c) Fi=13DM1N−M2      for i=4, 6, 7, 

(7d) F8=1DN−M3, 

(7e) D=N3,

with *N* = 3 (*n* − 3) for the 3-bit coding. As for the machine translations (red bars) in Figure 10a, in order from the left to the right, one can see Google Translate GT1 (as of December 2023; *χ*^2^ = 8.610), GT2 (as of May 2025; *χ*^2^ = 7.627), DeepL (as of July 2025; *χ*^2^ = 11.913), and GT3 (as of July 2025; *χ*^2^ = 4.429). As for the human translations (blue bars), of the 13 translations, only 2 on the right extreme, which correspond to Matsumura [42] (*χ*^2^ = 17.737 > 14.067) and Maruya [50] (*χ*^2^ = 49.610 > 24.321), are found to be statistically significant (*α* = 0.05 and 0.001, respectively). To quantify the robustness of our results for the 4-bit coding, a scattergram of the 3-bit coding versus the 4-bit counterpart is plotted in Figure 10b. It can be seen that the chi-square values between the two methods of coding exhibit strong positive correlation (*r* = 0.9699 with *d* = 1.751). To visualize the difference between the surveyed and expected frequency, in Figure 11, frequency distributions of the 3-bit binary codes *C_i_* (*i* = 1 to 8) are juxtaposed with red (surveyed) and navy (expected). Here, from Figure 10a the two bars on the left and right extremes are selected: (a) Akira Ooka [51] (*χ*^2^ = 1.134; *n* = 1084) and (b) Saiichi Maruya [50] (*χ*^2^ = 49.610; *n* = 1216). Similarly to the observation in Figure 6, it is found that the difference in the topography is consistent with that of the chi-square value. That is, in Figure 11b the sequence of the twin bars consisting of red and navy exhibits a rough topography in particular on the right, which shows a contrast to the relatively smooth topographic configuration being observed in Figure 11a.

**Figure 12:**
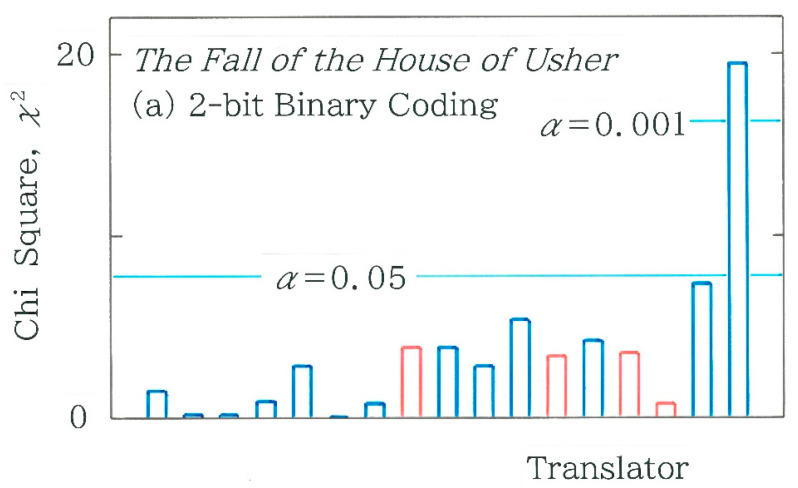

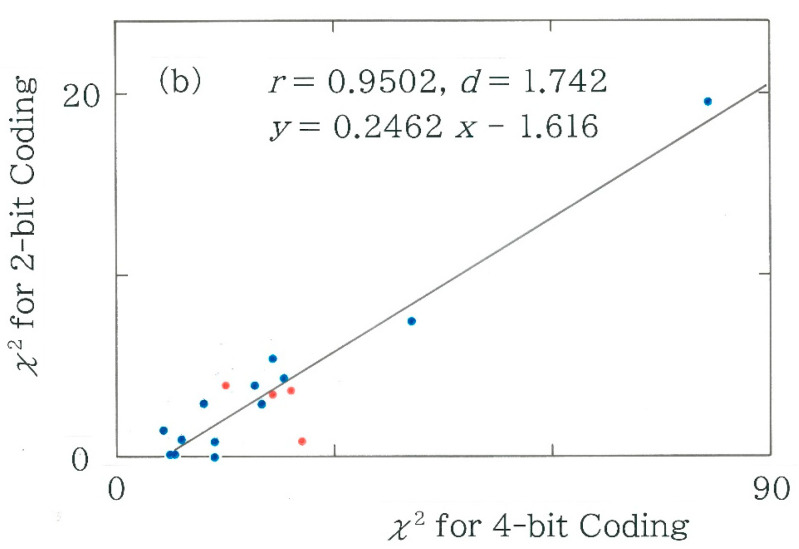
(**a**) Comparison among the chi-square values for 17 Japanese translations of the opening paragraph in *The Fall of the House of Usher* written by Edgar Allan Poe. The blue and red bars indicate the human and machine translations, respectively. The expected frequencies are calculated using Equation (4) along with Equations (8a)–(8d). (**b**) Scattergram of chi-square values for 2-bit coding versus those for 4-bit coding. The blue and red dots indicate the human and machine translations, respectively.

**Figure 13:**
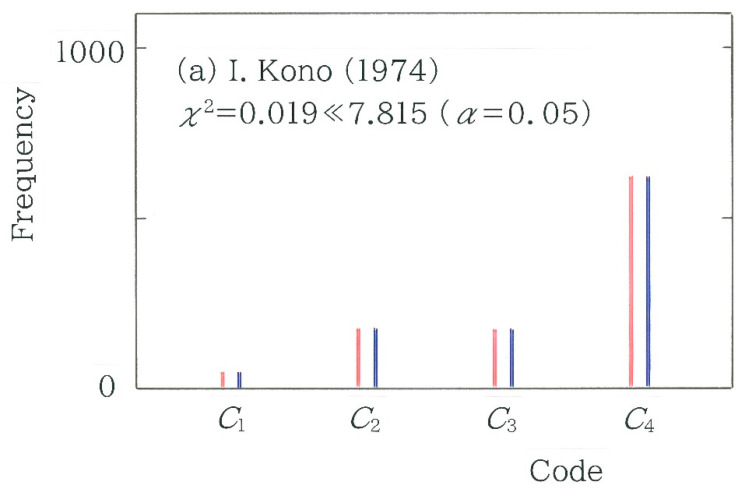

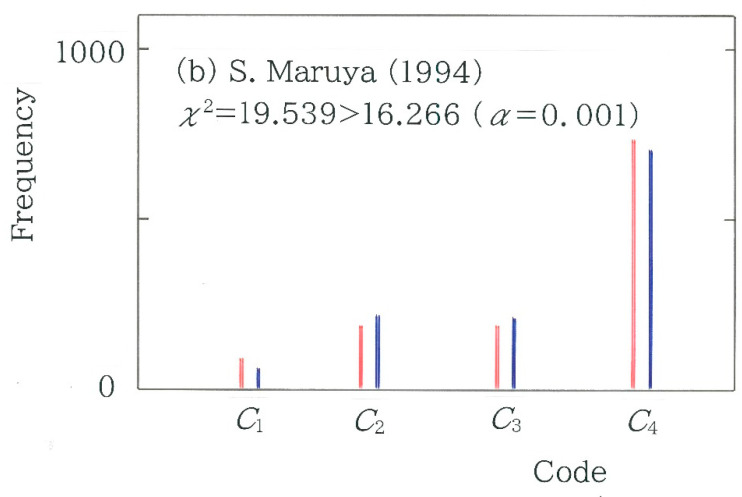
Frequency distributions of the 2-bit binary codes: *C*_1_ = 00, *C*_2_ = 01, *C*_3_ = 10, and *C*_4_ = 11. The red and navy bars indicate the surveyed and expected frequencies, respectively. (**a**) Translation by Ichiro Kono [47]. (**b**) Translation by Saiichi Maruya [50].

Lastly, in Figure 12, using the 2-bit binary coding, comparison will be made among chi-square values for 17 Japanese translations of the opening paragraph. The blue and red bars indicate the human and machine translations, respectively. In applying Equation (4) with *m* = 4 the expected frequencies *F_i_* (*i* = 1 to 4) for *C*_1_ = 00, *C*_2_ = 01, *C*_3_ = 10, and *C*_4_ = 11 are calculated with the amalgam of ‘0’ and ‘1’ in the entire binary sequence:

(8a) F1 =1DM2, 

(8b) Fi=12DM1N−M1      for i=2, 3, 

(8c) F4=1DN−M2,

(8d) D=N2, 

with *N* = 2 (*n* − 3) for the 2-bit coding. As for the machine translations (red bars) in Figure 12a, in order from the left to the right, one can see GT1 (*χ*^2^ = 3.900), GT2 (*χ*^2^ = 3.387), DeepL (*χ*^2^ = 3.580), and GT3 (*χ*^2^ = 0.798). As for the human translations (blue bars), of the 13 translations, a single bar on the right extreme, which corresponds to Maruya [50] (*χ*^2^ = 19.539 > 16.266), is statistically significant (*α* = 0.001). To quantify the robustness of the results for the 4-bit coding, a scattergram of the 2-bit coding versus the 4-bit counterpart is given in Figure 12b. Again, it can be seen that the chi-square values between the two coding schemes exhibit a strong positive correlation (*r* = 0.9502 with *d* = 1.742). To reveal the difference between the surveyed and expected frequency, in Figure 13, frequency distributions of the 2-bit binary codes *C_i_* (*i* = 1 to 4) are compared with red (surveyed) and navy (expected). Here, from Figure 12a the two bars on the left and right extremes are taken: (a) Ichiro Kono [47] (*χ*^2^ = 0.019; *n* = 1037) and (b) Saiichi Maruya [50] (*χ*^2^ = 19.539; *n* = 1216). Similarly to Figure 6 and Figure 11, it can be seen that the difference in the topography is consistent with that of the chi-square value.

## 6. Conclusions

We attempted to investigate the applicability of the ordinal pattern approach to analyzing the quality of texts that were translated into Japanese, which preserves the so-catted vowel harmony being inherent in Ural–Altaic languages. With the combination of the Hellinger distance as well as the chi-square statistic, computed results have been presented for the three cases: passages from a short novel by Kenzaburo Oe, the Preamble to the Constitution of Japan, and the 17 translations of the opening paragraph in a detective story by Edgar Allan Poe, including 13 human and 4 machine translations. A diachronic analysis of computed results for translations by humans and by artificial intelligence (AI) has shown that, in recent years, capabilities of one kind of AI appear to have been improved. However, for now, we still cannot affirm clearly that it has become a formidable competitor. Lastly, this paper has focused on a case study of a quantitative evaluation on the quality of both human and machine translations. There is systematic work ahead. To conclude, our methodology will be applicable also to other languages preserving a feature of vowel harmony, such as Ainu, Korean, Mongolian, Uighur, Telugu, Turkish, Hungarian, Finnish, Indonesian, and Swahili, all of which are expected to display an exquisite blend between the two binary symbols in the vowel sequences.

## Data Availability

The raw data supporting the conclusions of this article will be made available by the author without undue reservation.

## Appendix A. Published Translation of Japanese Passages from *Shiiku* into English [38]

From the cluster of grown-ups my father stepped forward, a hatchet in his hand. His eyes were ablaze with anger, hot like those of a dog. The ruffian’s nails bit deeper into my neck, and I moaned. Father rushed at us brandishing the hatchet above his head. I shut my eyes. Grasping my left wrist, the ruffian pulled my arm up to protect his head. A howl rose from the crowd assembled in the cellar, and I heard the smashing of my left hand and the ruffian’s skull. On the oily, shiny skin of the ruffian’s arm beneath my chin the blood splashed down in thick globules. The grown-ups rushed towards us, and simultaneously I felt the slackening of the ruffian’s arm and a burning pain throughout my body.

## Appendix B. The English Version of the Permeable to the Constitution of Japan

We, the Japanese people, acting through our duly elected representatives in the National Diet, determined that we shall secure for ourselves and our posterity the fruits of peaceful cooperation with all nations and the blessings of liberty throughout this land, and resolved that never again shall we be visited with the horrors of war through the action of government, do proclaim that sovereign power resides with the people and do firmly establish this Constitution. Government is a sacred trust of the people, the authority for which is derived from the people, the powers of which are exercised by the representatives of the people, and the benefits of which are enjoyed by the people. This is a universal principle of mankind upon which this Constitution is founded. We reject and revoke all constitutions, laws, ordinances, and rescripts in conflict herewith.

We, the Japanese people, desire peace for all time and are deeply conscious of the high ideals controlling human relationship, and we have determined to preserve our security and existence, trusting in the justice and faith of the peace-loving peoples of the world. We desire to occupy an honored place in an international society striving for the preservation of peace, and the banishment of tyranny and slavery, oppression and intolerance for all time from the earth. We recognize that all peoples of the world have the right to live in peace, free from fear and want.

We believe that no nation is responsible to itself alone, but that laws of political morality are universal; and that obedience to such laws is incumbent upon all nations who would sustain their own sovereignty and justify their sovereign relationship with other nations.

We, the Japanese people, pledge our national honor to accomplish these high ideals and purposes with all our resources.

## Appendix C. The Opening Paragraph Extracted from *The Fall of the House of Usher* by Edgar Allan Poe [40]

During the whole of a dull, dark, and soundless day in the autumn of the year, when the clouds hung oppressively low in the heavens, I had been passing alone, on horseback, through a singularly dreary tract of country, and at length found myself, as the shades of the evening drew on, within view of the melancholy House of Usher. I know not how it was―but, with the first glimpse of the building, a sense of insufferable gloom pervaded my spirit. I say insufferable, for the feeling was unrelieved by any of that half-pleasurable, because poetic, sentiment, with which the mind usually receives even the sternest natural images of the desolate or terrible. I looked upon the scene before me―upon the mere house, and the simple landscape features of the domain―upon the bleak walls―upon the vacant eye-like windows―upon a few rank sedges―and upon a few while trunks of decayed trees―with an utter depression of soul which I can compare to no earthly sensation more properly than to the after-dream of the reveler upon opium―the bitter lapse into everyday life―the hideous dropping off of the veil. There was an iciness, a sinking, a sickening of the heart―an unredeemed dreariness of thought which no goading of the imagination could torture into aught of the sublime. What was it―I paused to think―what was it that so unnerved me in the contemplation of the House of Usher? It was a mystery all insoluble; nor could I grapple with the shadowy fancies that crowded upon me as I pondered. I was forced to fall back upon the unsatisfactory conclusion that while, beyond doubt, there are combinations of very simple natural objects which have the power of thus affecting us, still the analysis of this power lies among considerations beyond our depth. It was possible, I reflected, that a mere different arrangement of the particulars of the scene, of the details of the picture, would be sufficient to modify, or perhaps to annihilate, its capacity for sorrowful impression; and, acting upon this idea, I reined my horse to the precipitous brink of a black and lurid tarn that lay in unruffled luster by the dwelling, and gazed down―but with a shudder even more thrilling than before―upon the remodeled and inverted images of the gray sedge, and the ghastly tree stems, and the vacant and eye-like windows.
